# Supplementary material for: In vivo cell type-specific CRISPR knockdown of dopamine beta hydroxylase reduces locus coeruleus evoked wakefulness
Source: Nat Commun. 2018 Dec 6;9:5211. doi: 10.1038/s41467-018-07566-3 (PMC6283864; doi:10.1038/s41467-018-07566-3)
Supplement: Supplementary file 1 — Supplementary Information [file 41467_2018_7566_MOESM1_ESM.pdf]

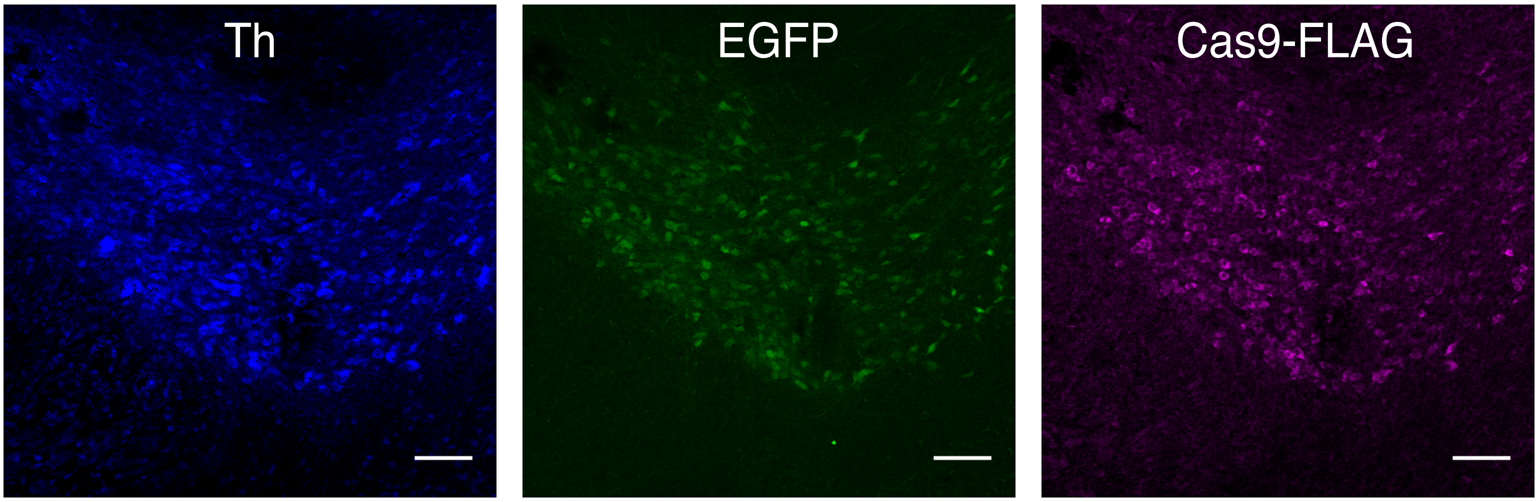

Supplementary Figure 1. Representative immunofluorescence images of Th, EGFP and Cas9-FLAG in the ventral tegmental area from Th/Cas9 mice. Scale bar, 100μm

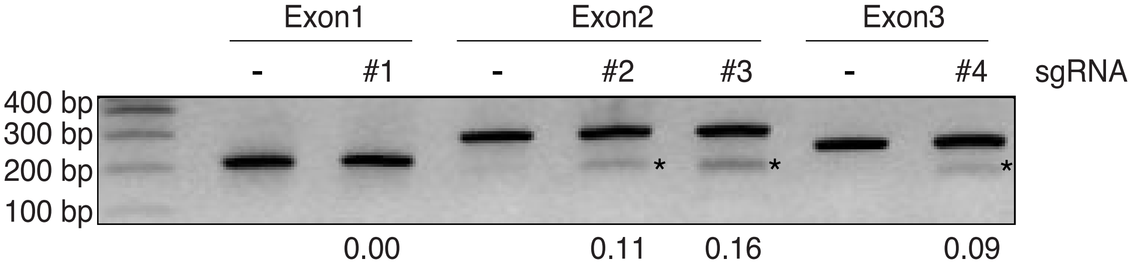

Supplementary Figure 2. Agarose gel electrophoresis of T7 endonuclease I-treated PCR products derived from NIH3T3 cells trasfected with sgRNAs and Cas9. The numbers indicate the ratio of cleaved vs uncleaved PCR products.

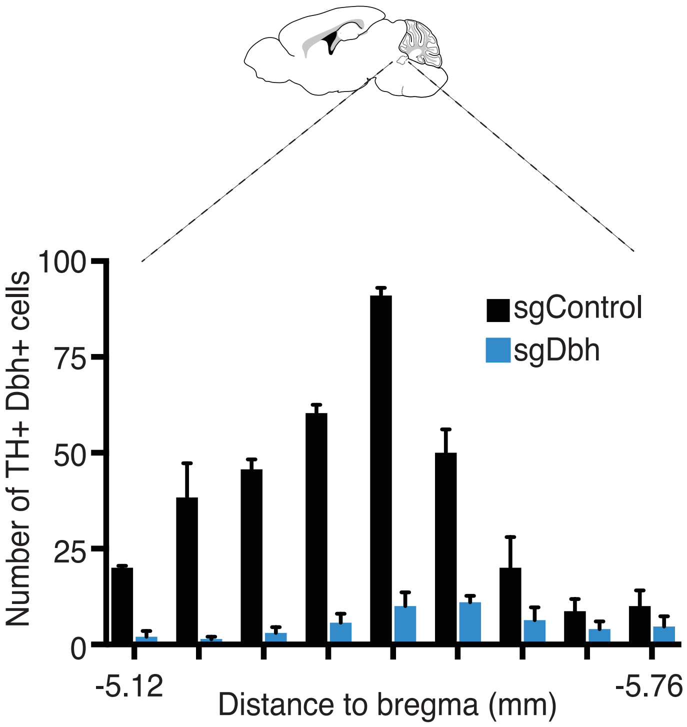

Supplementary Figure 3. (a) Quantification of Dbh and Th-double positive cells in LC neurons from Th/Cas9 mice injected with AAV-DJ sgDbh or AAV-DJ sgControl in 40 μm brain sections from the rostral-to-caudal ends of the LC (anteroposterior, –5.12 to –5.76). Cell counts are plotted as mean ± s.e.m (n = 3 mice). The LC was defined by the Allen Reference Atlas[26].

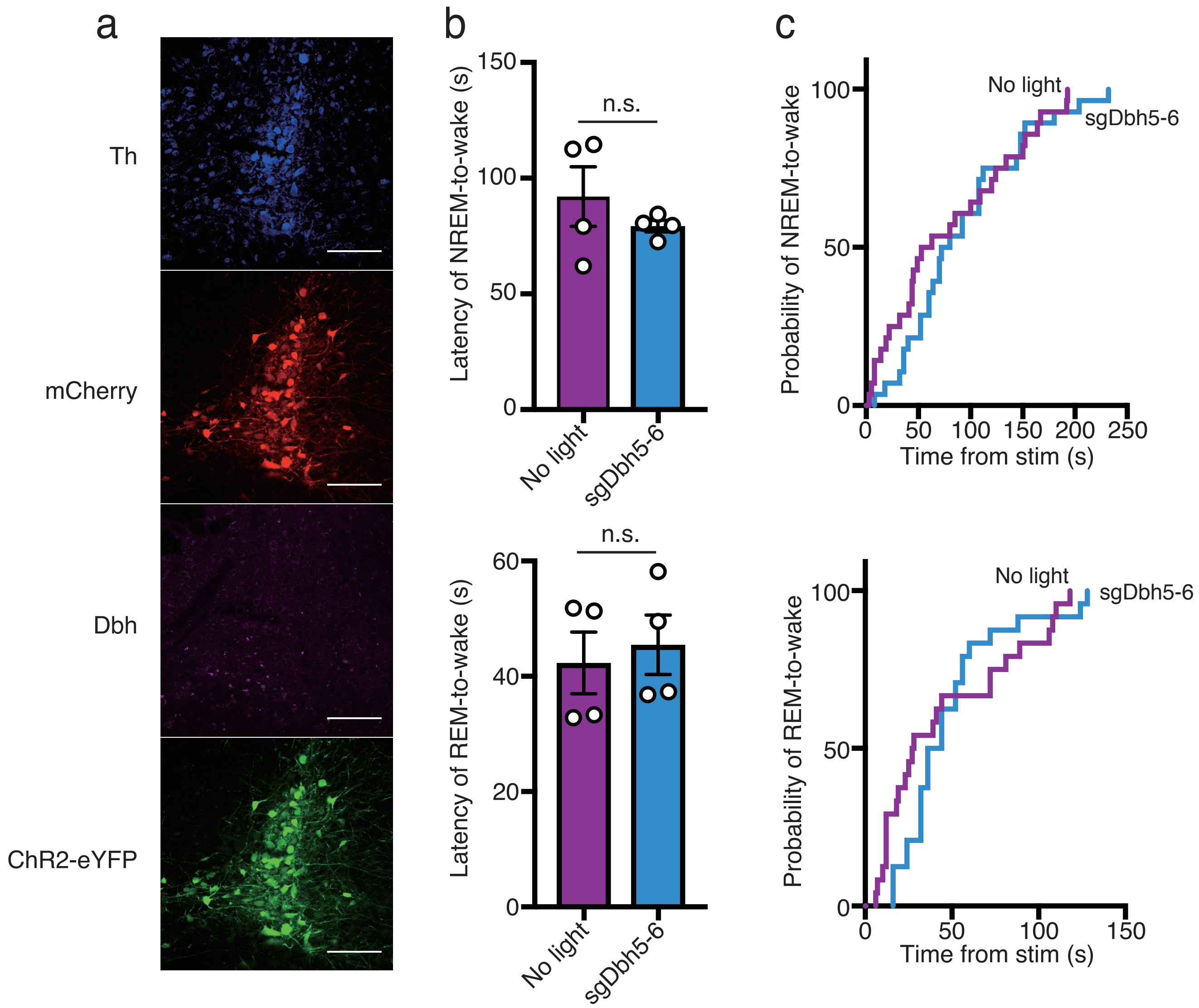

Supplementary Figure 4. The disruption of dbh gene using re-designed sgRNAs.(a) Representative immunofluorescence images of the LC from Th/Cas9 mice injected with AAV-DJ sgDbh5-6 together with AAV-DJ DIO ChR2. Scale bar, 100 μm. (b) Latencies (mean±s.e.m) to wake during NREM (top) or REM (bottom) sleep following the unilateral photostimulation (10-ms pulses at 20 Hz for 10 s) of LC neurons in a Th/Cas9 mouse injected with AAV-DJ sgDbh together with AAV-DJ DIO ChR2 (n = 4; mean latency of 6-7 stimulations per each mice are plotted) (not significant (n.s.) by two-tailed Student t-test between No light and sgDbh5-6; p = 0.3680 (NREM) and 0.6883 (REM) t = 0.9702 (NREM) and 2.382 (PFC), df = 6 (NREM) and 6 (REM)) (c) Cumulative distribution of sleep-to-wake transitions following the unilateral photostimulation (10-ms pulses at 20 Hz for 10 s) of LC neurons during NREM (top) or REM sleep (bottom).

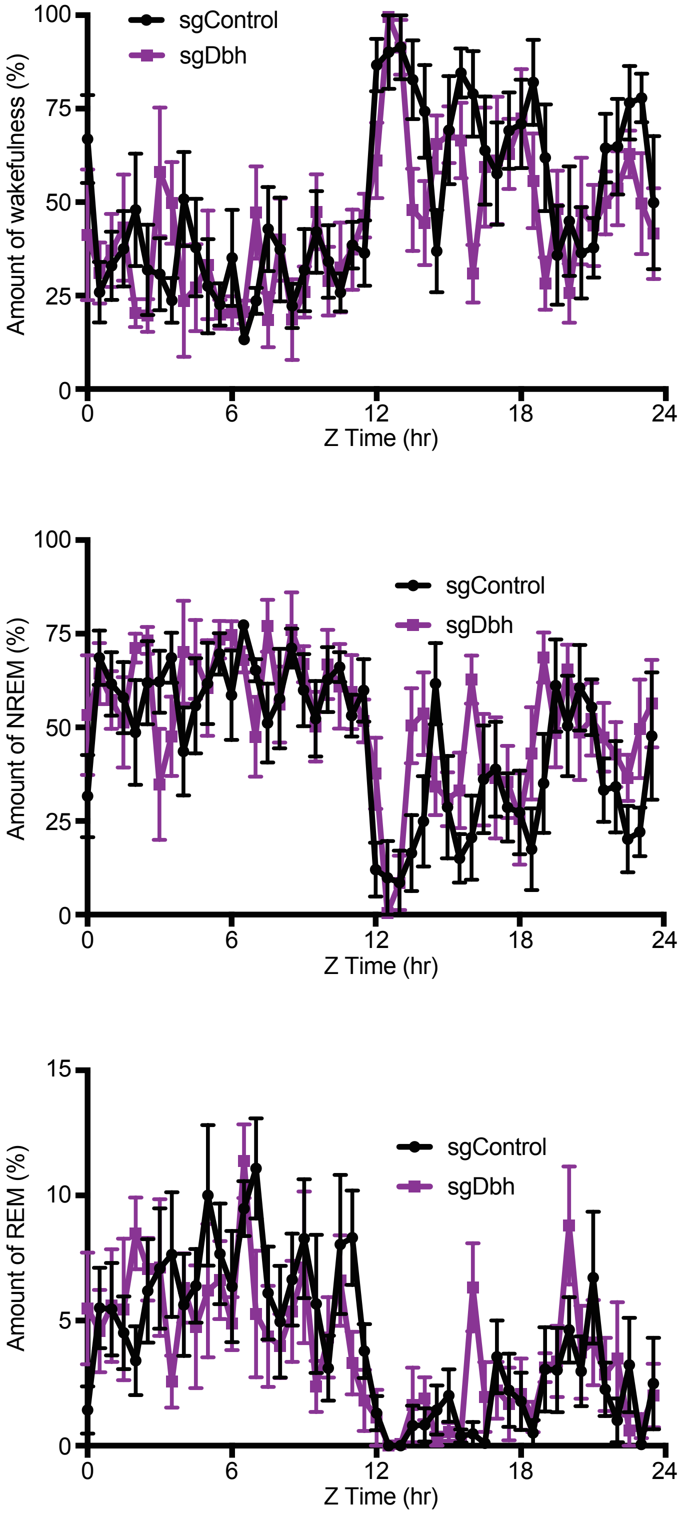

Supplementary Figure 5. Percent (mean ± s.e.m) time spent in wakefulness (top), NREM (middle) or REM (bottom) sleep during 24 h in Control and sgDbh-treated mice.
